# Agent-based modelling of *Mycobacterium tuberculosis* transmission: a systematic review

**DOI:** 10.1186/s12879-024-10245-y

**Published:** 2024-12-06

**Authors:** Viet Long Bui, Angus E. Hughes, Romain Ragonnet, Michael T. Meehan, Alec Henderson, Emma S. McBryde, James M. Trauer

**Affiliations:** 1 School of Public Health and Preventive Medicine, Monash University, Melbourne, VIC Australia; 2 Australian Institute of Tropical Health and Medicine, James Cook University, Townsville, QLD Australia

**Keywords:** Agent-based modelling, Tuberculosis, Mycobacterium tuberculosis, Transmission

## Abstract

**Background:**

Traditional epidemiological models tend to oversimplify the transmission dynamics of *Mycobacterium tuberculosis (M.tb)* to replicate observed tuberculosis (TB) epidemic patterns. This has led to growing interest in advanced methodologies like agent-based modelling (ABM), which can more accurately represent the complex heterogeneity of TB transmission.

**Objectives:**

To better understand the use of agent-based models (ABMs) in this context, we conducted a systematic review with two main objectives: (1) to examine how ABMs have been employed to model the intricate heterogeneity of M.tb transmission, and (2) to identify the challenges and opportunities associated with implementing ABMs for M.tb.

**Search methods:**

We conducted a systematic search following PRISMA guidelines across four databases (MEDLINE, EMBASE, Global Health, and Scopus), limiting our review to peer-reviewed articles published in English up to December 2022. Data were extracted by two investigators using a standardized extraction tool. Prospero registration: CRD42022380580.

**Selection criteria:**

Our review included peer-reviewed articles in English that implement agent-based, individual-based, or microsimulation models of *M.tb* transmission. Models focusing solely on in-vitro or within-host dynamics were excluded. Data extraction targeted the methodological, epidemiological, and computational characteristics of ABMs used for TB transmission. A risk of bias assessment was not conducted as the review synthesized modelling studies without pooling epidemiological data.

**Results:**

Our search initially identified 5,077 studies, from which we ultimately included 26 in our final review after exclusions. These studies varied in population settings, time horizons, and model complexity. While many incorporated population heterogeneity and household structures, some lacked essential features like spatial structures or economic evaluations. Only eight studies provided publicly accessible code, highlighting the need for improved transparency.

**Authors’ conclusions:**

ABMs are a versatile approach for representing complex disease dynamics, particularly in cases like TB, where they address heterogeneous mixing and household transmission often overlooked by traditional models. However, their advanced capabilities come with challenges, including those arising from their stochastic nature, such as parameter tuning and high computational expense. To improve transparency and reproducibility, open-source code sharing, and standardised reporting are recommended to enhance ABM reliability in studying epidemiologically complex diseases like TB.

**Supplementary Information:**

The online version contains supplementary material available at 10.1186/s12879-024-10245-y.

## Introduction

Tuberculosis (TB), caused by the bacterium *Mycobacterium tuberculosis (M.tb)*, remains a significant global health concern, ranking among the top causes of mortality worldwide [1]. While compartmental models have often been used to guide TB control strategies, they may not fully capture the complexity and heterogeneity of TB transmission dynamics [2]. In recent years, agent-based modelling (ABM) has become an increasingly popular approach for modelling infectious diseases due to its capacity to capture the complexity of human interactions and behaviours that contribute to disease transmission [3]. ABMs allow researchers to simulate individual-level behaviours and interactions, providing insights into how disease spreads through a population over time. ABMs have been used to model a range of infectious diseases, including seasonal influenza, malaria, Ebola and [4–7], recently COVID-19 [8–11].

TB possesses distinctive attributes that strongly influence the optimal choice of modelling framework. The age-dependent transition rates from latent TB infection (LTBI) to active disease underline the significance of incorporating age-related structures into TB models. Moreover, the concentration of *M.tb* transmission within high-risk population clusters accentuates the need to factor in heterogeneous social mixing within the population as part of the framework. This is an important consideration when attempting to identify unobserved transmission patterns [12].

The substantial impact of various coexisting conditions, notably HIV, on *M.tb* transmission often necessitates their inclusion within the model’s architecture. There may also be considerable interplay between medical comorbidities, which may be difficult to capture with compartmental models. Additionally, acknowledging the lifelong persistence of reactivation risk following infection may necessitate an extended temporal perspective to fully capture the disease’s trajectory over many decades [13].

Agent-based models (ABMs) excel in explicitly simulating population heterogeneity, encompassing individual traits, behaviours, contact dynamics, interpersonal networks, and location specific transmission, such as households [3]. This attribute of ABMs holds particular relevance for infectious diseases like TB. By representing individuals as discrete agents with diverse characteristics and interactions, ABMs can amalgamate these features to capture *M.tb* transmission within households and across broader community settings.

Leveraging these advanced modelling methodologies can permit the identification of pockets of high-risk populations and elucidate their roles in transmission dynamics. Consequently, the ABM framework can facilitate the assessment of targeted interventions and inform effective TB control and prevention strategies.

We reviewed evidence on the use of ABMs in modelling the transmission of *M.tb*. We addressed two main questions: (1) How have ABMs been utilized to model the complex heterogeneity of *M.tb* transmission? and (2) What are the challenges and opportunities in implementing ABMs for *M.tb*? By addressing these questions, we aimed to enhance understanding of the role ABMs play in modelling M.tb transmission dynamics and provide insights for future research in this area.

## Methods

### Search strategy

Our search, extraction and reporting strategy was based on the PRISMA protocol (Preferred Reporting Items for Systematic Reviews and Meta-Analyses) and Cochrane guidelines [14] (see Appendix 1 and Appendix 2 for PRISMA checklists). We prospectively registered our review protocol with International Prospective Register of Systematic Reviews (PROSPERO, registration number: CRD42022380580), available at: https://www.crd.york.ac.uk/prospero/display_record.php?RecordID=380580.

We identified publications from four electronic databases (MEDLINE, EMBASE, Global Health, and Scopus) using predesignated search terms. We used ABM as our main search term for identifying models that simulated populations at the individual level but also captured studies with terms including individual-based models (IBMs) and microsimulations. The search strategy was designed to identify publications that mentioned each of the following three concepts in their subject headings, keywords, titles, or abstracts:

1. Terms relating to *M.tb;*
2. Terms relating to epidemiology, disease outbreaks or transmission, and epidemics;
3. Terms identifying ABMs, individual-based or microsimulation models.

To ensure comprehensive coverage, we combined terms from each category using the Boolean OR operator and linked these groupings with the Boolean AND operator to refine searches across titles, keywords, and abstracts in selected databases. A detailed explanation of our search strategy can be found in Appendix 3.

### Study selection

We hierarchically considered full-text articles against our inclusion and exclusion criteria to determine eligibility, restricting our review to peer-reviewed articles published in English up until December 2022. We imposed no geographical restrictions on the studies included. The abstracts of all retrieved studies were reviewed for relevance, specifically selecting those that discussed an agent-based, individual-based or microsimulation model of *M.tb* transmission for full-text evaluation. Models focusing solely on in-vitro or within-host dynamics were excluded.

Search results were exported to EndNoteX9 (Clarivate Analytics, NY, USA) and duplicates were removed. We used 3-stage screening to identify studies for inclusion: Stage 1 (Title review): two reviewers VLB and AEH independently screened titles. If a title was clearly irrelevant, the article was dropped; if it was selected by either reviewer, it progressed to the next stage; Stage 2 (Abstract review): two reviewers (VLB and AEH) independently reviewed abstracts. Studies progressed to the next stage of review if both reviewers agreed on inclusion; Stage 3 (Full-text review): two reviewers (VLB and AEH) independently assessed the full-text article. If there was disagreement, reviewers met to review and reach consensus on whether to include or exclude the studies. If reviewers still disagreed, a third reviewer (JMT) was available to resolve differences, although this was not needed.

### Data collection and analysis

Data were extracted using a spreadsheet developed, tested and approved by two investigators (LB, AH). We extracted various characteristics of the agent-based models (ABMs) used in *M.tb* TB research. These characteristics encompassed whether the model represented the entire population, the TB burden level (high, moderate, or low) as reported by the authors, age representation of agents, heterogeneous mixing among individuals, inclusion of LTBI, considered interventions, representation of household structure, inclusion of spatial structure, presence of an economic evaluation, the type of software used (whether simulation or self-developed), and the availability of the model code.

### Assessment of quality

We did not conduct a risk of bias assessment because the review focused on synthesizing modelling studies without pooling epidemiological data, where such assessments are typically more relevant.

## Results

**Fig. 1:**
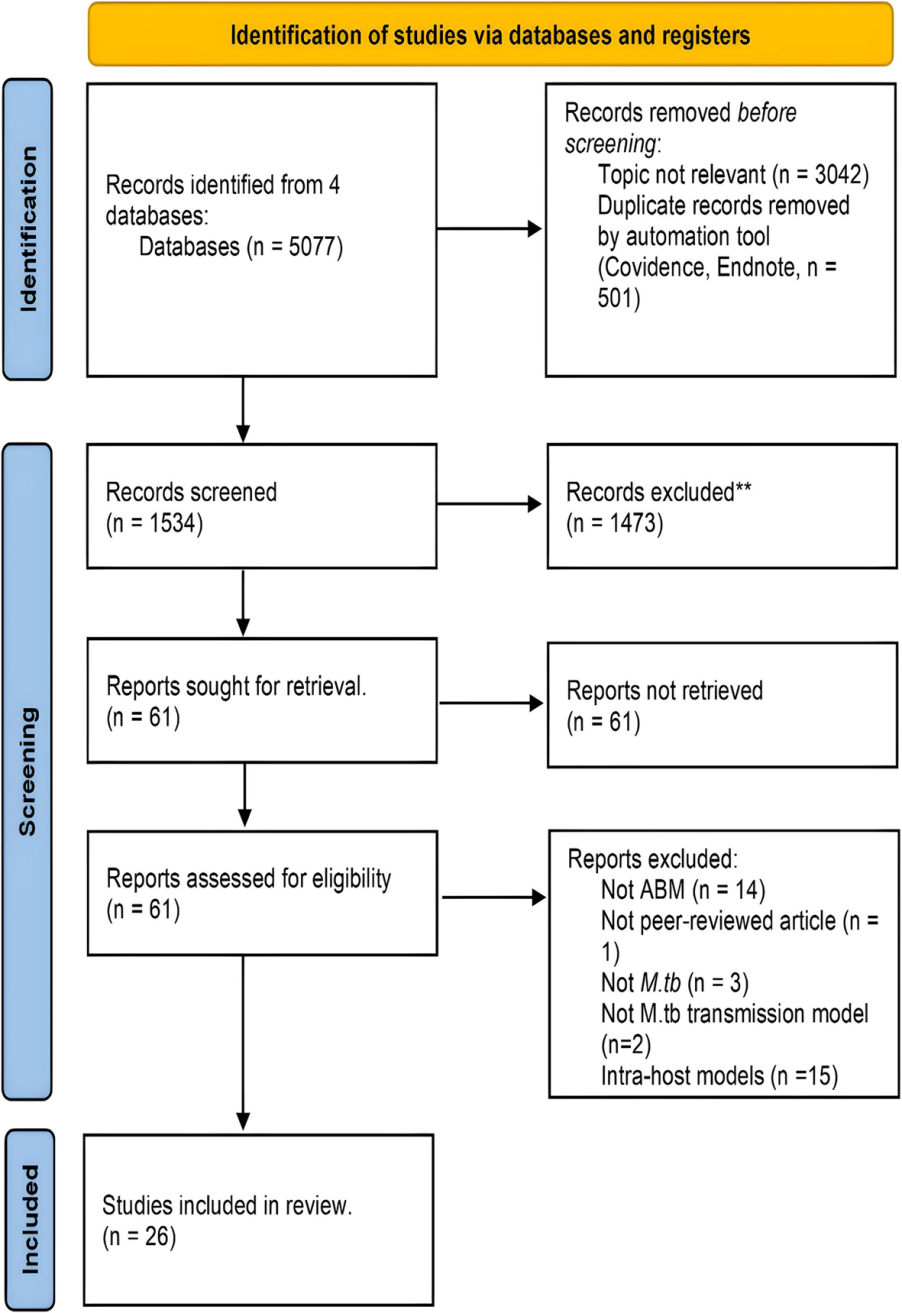
PRISMA 2020 flow diagram for systematic reviews which included searches of databases and registers

 Our search strategy identified 5077 studies, and we assessed these papers’ general attributes. Prior to the review, 3543 records were excluded for duplicate or irrelevant themes. 1534 records were reviewed, with 1435 rejected based on title and abstract screening. Sixty-one records progressed to full-text screening, with 26 included in our final review (Fig. 1). Table 1 presents the list of included studies, while Table 2 and Appendix 4 provide detailed descriptions of the key epidemiological and methodological characteristics of the 26 reviewed studies.

**Table 1:** Included studies

No	Title	Year of publication	Citation
1	Are survey-based estimates of the burden of drug resistant TB too low? Insight from a simulation study	2008	[15]
2	An agent-based computational model of the spread of tuberculosis	2011	[16]
3	Modeling socio-demography to capture tuberculosis transmission dynamics in a low-burden setting	2011	[17]
4	Evaluating the effectiveness of contact racing on tuberculosis outcomes in Saskatchewan using individual-based modeling	2013	[18]
5	Can Australia eliminate TB? Modelling immigration strategies for reaching MDG targets in a low-transmission setting	2014	[19]
6	Disease control implications of India’s changing multidrug-resistant tuberculosis epidemic	2014	[20]
7	Timing of tuberculosis transmission and the impact of household contact tracing: An agent-based simulation model	2014	[21]
8	A novel tool improves existing estimates of recent tuberculosis transmission in settings of sparse data collection	2015	[22]
9	An agent-based computational model for tuberculosis spreading on age-structured populations	2015	[23]
10	Cost-effectiveness of improvements in diagnosis and treatment accessibility for tuberculosis control in India	2015	[24]
11	Tuberculosis control strategies to reach the 2035 global targets in China: the role of changing demographics and reactivation disease	2015	[25]
12	Individual-based modeling of tuberculosis in a user-friendly interface: Understanding the epidemiological role of population heterogeneity in a city	2016	[26]
13	Comparing drivers and dynamics of tuberculosis in California, Florida, New York, and Texas	2017	[27]
14	Stochastic agent-based modeling of tuberculosis in Canadian Indigenous communities	2017	[28]
15	An explanation for the low proportion of tuberculosis that results from transmission between household and known social contacts	2018	[29]
16	Small contribution of gold mines to the ongoing tuberculosis epidemic in South Africa: A modeling-based study	2018	[30]
17	Impact and effectiveness of state-level tuberculosis interventions in California, Florida, New York, and Texas: A model-based analysis	2019	[31]
18	Outlook for tuberculosis elimination in California: An individual-based stochastic model	2019	[32]
19	Profiling *Mycobacterium tuberculosis* transmission and the resulting disease burden in the five highest tuberculosis burden countries	2019	[33]
20	A framework for network-based epidemiological modeling of tuberculosis dynamics using synthetic datasets	2020	[34]
21	Modeling the impact of recommendations for primary care-based screening for latent tuberculosis infection in California	2020	[35]
22	Tuberculosis from transmission in clinics in high HIV settings may be far higher than contact data suggest	2020	[36]
23	Modelling the effect of infection prevention and control measures on rate of Mycobacterium tuberculosis transmission to clinic attendees in primary health clinics in South Africa	2021	[37]
24	Representing tuberculosis transmission with Complex Contagion: An agent-based simulation modeling approach	2021	[38]
25	Estimating the contribution of transmission in primary healthcare clinics to community-wide TB disease incidence, and the impact of infection prevention and control interventions, in KwaZulu-Natal, South Africa	2022	[39]
26	Evaluation of TB elimination strategies in Canadian Inuit populations: Nunavut as a case study	2022	[40]

**Table 2:** Characteristics of the reviewed studies

**Characteristics**	**N**	**%**
**TB Burden**^**1**^		
High	11	42%
Moderate	1	4
Low	12	46%
Unclear	2	8%
**Heterogeneous mixing**		
Yes	15	58%
No	11	42%
**Age explicitly modelled**		
Yes	12	46%
No	14	54%
**Household structure represented**		
Yes	8	31%
No	18	69%
**Latent TB represented**		
Yes	23	88%
No	3	12%
**Spatial structure**		
Yes	5	18%
No	21	82%
**Economic evaluation**		
Yes	2	8%
No	24	92%
**Software**		
Simulation software^2^	12	50%
Self-developed^3^	9	32%
Not Declared	5	18%
**Code publicly accessible**		
Yes	8	29%
No	18	71%
**Total**	26	100%

^1^As reported by authors

^2^AnyLogic, NetLogo, EMOD, DTK)

^3^MATLAB^®^, C/C++, Python, Go, Julia, etc.…

### Analysis objectives

The overarching purpose of the included studies was typically described as being either to capture and understand the underlying transmission dynamics [8, 9], or to evaluate the effectiveness of different TB interventions. Interventions of interest included directly observed therapy short-course, contact tracing, active case detection, latent TB infection (LTBI) testing and treatment [10–13].

### Population and settings

Out of 26 included studies, 11 studies representing transmission in high-burden countries [15–25], 12 studies simulating low-burden countries [26–37], 2 studies unclear [38, 39], only 1 study in a moderate setting. The 26 studies had simulated model population sizes ranging between 3,786 people [36] to more than 6 million people [22].

To represent the sizes of the target populations, several investigators utilised scale factors. A scale factor refers to a parameter that relates the modelled population size to the real-world population it is intended to represent. By adjusting the scale factor, researchers can reduce the number of agents or individuals required, striking a balance between capturing essential dynamics and minimising computational requirements. For instance, Chang et al. used multiple scale factors to model people in each of the various groupings, scaling down in the model the persons in the mining, peri-mining, and labour-sending categories by a factor of around 50 [20]. To reduce the computational expense, Ragonnet et al. modelled only a fraction of the national population of each simulated country [22], while Goodell et al. used a scale factor of 1000 [28].

### Natural history of infection and disease

ABMs commonly incorporate health states or compartments to represent stages in the natural history of disease similar to those found in traditional compartmental models, such as the traditional susceptible - exposed - infected - recovered (SEIR) model [40]. In an ABM employing the SEIR paradigm, agents were categorised into one of four states according to their status regarding the pathogen of interest: susceptible (S), exposed (E), infectious (I), and recovered (R). Rules that govern disease transmission then dictated how the individual agents interacted. ABMs often divided the latent period between *M.tb* infection and subsequent TB disease into early and late phases to capture the observed declining risk of disease with time from infection (23 out of 26) [11–14, 16–22, 24, 26–30, 32, 34, 36, 38, 39, 41]. These models commonly utilised data derived from the published literature or public datasets to set estimates for input parameters. While calibration techniques were commonly used to estimate free model parameters, only one of the included studies reported validating parameters that had been tuned using an independent dataset [26].

### Heterogeneous mixing and household structure

Fifteen out of 26 included studies implemented some form of heterogeneous social mixing, although a variety of approaches were used [15, 20–22, 25–27, 32, 35–37]. Only 8 out of 26 studies explicitly incorporated household structure [21, 22, 25, 26, 32, 35, 37, 41]. Two studies utilised setting-specific contact data [25, 37], while other authors used synthetic population data [13, 39]. Tian et al. used the Barabasi and Albert algorithm to determine the shape of the contact network [42], while Tuite et al. included age-assortative mixing within households and communities [32]. The household sizes in the model of Kasaie et al. were varied to reflect underlying population household size but without specifically taking the age structure of the household into account [41]. Cohen et al. used a distance function that could be varied to modify network connectivity [15], while Guzzetta et al. used a heuristic contact network to replicate household structures [34]. McCreesh et al. (2018) focused specifically on household transmission but found that while it contributed substantially to TB epidemiology, saturation of contacts and the influence of “superspreaders’’ at locations outside households limited its impact on transmission overall [21].

Only five of the included studies incorporated a spatial component. Prats et al. used a spatial approach by randomly generating a population based on input parameter distributions, which were then distributed across a 501× 501 grid [31]. Zwick et al. allowed agents to move between grids in their respective neighbourhoods, where contacts were made between agents within each grid space and its adjoining spaces [36]. Meanwhile, Guzzetta et al. allocated the population to a spatial grid based on observed spatial population density [26], while the contact matrix of Cohen et al. allowed for the probability of an edge (and so risk of transmission) between two individuals to decrease with increasing spatial separation.

Comorbidities, such as HIV [37], can also be implemented as characteristics of agents. In the model developed by Chang [20], HIV exerted a substantial influence by elevating the rate of disease activation across all groups, including mine workers. The relationship between HIV presence and increased latent-to-active TB risk underscores the need to consider HIV comorbidity in understanding TB dynamics. This is particularly pertinent when HIV prevalence is high within the simulated population and significantly influences transmission patterns.

### Economic evaluation

Among 26 studies, only four conducted economic evaluations. Goodell et al.‘s study assessed LTBI testing and treatment in California, projecting 106,000 TB cases in the state by 2065. Pre-elimination could be achieved by 2065 under certain scenarios, with costs ranging from $20B to $48B and incremental cost-effectiveness ratios (ICERs) from $657,000 to $3.1 M per QALY [28]. Reddy’s study in Malawi and South Africa showed cost-effective interventions increasing life expectancy by 0.5–1.2 years, with an incremental ICER of $45 per year of life saved (YLS) and $840/YLS and decreased over time [43]. Suen’s study revealed that PPM, either alone or combined with Xpert diagnostics, was cost-effective, with benefits exceeding 1 GDP per capita per quality-adjusted life-year gained, surpassing Xpert interventions without PPM [18].

### Time horizons and time steps

A wide range of time horizons were used by included studies, ranging from 2 months [31] to 205 years [32]. The step sizes of the models ranged from just under one hour [27] to one year [22, 30]. Several papers explicitly reported the presence of a burn-in or warm-up period [19, 26, 27, 32].

### Software/Language

We categorised studies into two groups according to the software used: pre-existing simulation software and self-developed software. Within the first group, studies utilised various dedicated modelling platforms for ABM implementation, such as NetLogo [24, 25, 31], AnyLogic [24, 32], EMOD [20], and CEPAC-I [31]. In the other group, nine authors adopted the alternative approach of creating custom ABM platforms using tools that included mathematical software like MATLAB^®^ and other general-purpose programming languages such as Python, C/C++, and Java [11, 14, 16, 17, 22, 26, 27, 29, 30, 32, 34, 39, 41]. Only 8 of these studies made their code repositories available for access.

We note that certain technical information or features may have been absent from the published materials, particularly if model code was not provided as open-source or explained elsewhere. Additionally, in certain articles, it was necessary to extract information from the “Results” or “Discussion” sections as well as the figure captions in order to infer the model’s properties (such as population size, time horizon, step size, or the number of realisations).

While ABMs are a relatively recent addition to TB and infectious disease modelling, there are established frameworks, like ODD (Overview, Design concepts, and Details), that have been created to facilitate the documentation of models for publication. It’s worth noting that only Prats has thus far documented their model following the ODD framework [31].

### Number of realisations and model calibration

In the case of stochastic ABMs, a single set of initial conditions and input parameters can lead to different outcomes, such that multiple realisations are popular approach The number of realisations for each parameter set used to quantify the uncertainty in the results varied in our search from 100x up to 1600x. Guzzetta 2011 used a Nelder-Mead gradient descent algorithm to identify the best parameter sets [26], while Latin Hypercube Sampling was employed by 2 included studies [35, 41]. For the remaining 16 papers, we were unable to retrieve the number of realisations used.

### Computational cost

A minority of included studies reported the computational performance of their models. According to Renardy et al., their simulation runs were performed using a laptop with an Intel Core i7 processor clocked at 3.1 GHz and 16GB of 2133 MHz LPDDR3 RAM [35]. They observed that a single simulation over a 2-year timeframe on this device required roughly five minutes of CPU time to complete. In the ABM of Ragonnet et al., a full simulation lasting 205 years was completed in roughly three hours using an Intel Xeon E3-12xx v2 CPU (3.1 GHz, 8 MB Cache) [22].

## Discussion

Our review identified 26 articles that applied various agent-based modelling frameworks across many different scenarios, settings, and populations, characterised by varying sizes, geographical locations, and TB burdens. In contrast to the study of Lander et al. [3] which focused on the rise of individual-based models (IBMs) of infectious diseases, our review focused on the diverse application of ABMs to various scenarios, but explicitly for TB transmission. Despite the widely varied capacities of ABMs, the primary justification for choosing this approach was frequently not explicitly stated by the authors. In those that did justify their approach, a key aspect emphasised in the majority of analyses was the inclusion of heterogeneous mixing. This showcases ABMs’ potential to capture the local complexities of disease transmission dynamics relevant to TB epidemiology.

Included ABMs often adopted an SEIR framework to represent TB disease states, with agents assigned to different states based on their health status with regard to TB infection. While some studies divided the period of latent infection into early and late phases to capture the declining risk of TB disease over time, the flexibility of ABMs provides the advantage of being able to assign any distribution of sojourn times, such that this dichotomy is not essential. Yet, the importance of this advantage is uncertain, due to the limited detailed epidemiological understanding of TB latency dynamics.

Input parameters to these models were often derived from published literature, with data obtained from epidemiological datasets. However, only one included study validated the calibration algorithm with a separate dataset (i.e. distinct from the “training” dataset). This suggests that while calibration techniques were frequently used to identify optimal model parameters, validation using independent datasets was less prevalent.

While the potential of ABMs to capture the complexities of heterogeneous mixing is widely recognized, the integration of this crucial aspect remains limited in the reviewed studies. This disparity between the acknowledged importance of heterogeneous mixing and its inclusion in a relatively small proportion of studies suggests a significant gap in fully leveraging the capabilities of ABMs for modelling TB dynamics. The underrepresentation of heterogeneous mixing in the majority of studies raises questions about the extent to which the dynamics of real-world interactions and disease transmission are accurately represented.

Heterogeneous population mixing encompasses various interaction patterns among individuals in a population, accounting for differences in social behaviours, activity levels and contact patterns, all of which exert a substantial influence on disease transmission dynamics. In real-world systems, transmission is not distributed uniformly across space and time, but rather is clustered in specific locations or periods. When spatial heterogeneity in transmission is accurately represented, these models hold the potential to replicate transmission hotspots, and so quantify their importance to TB epidemiology. Furthermore, households are key in transmitting infectious diseases like TB [37], as close and prolonged contacts within them facilitate disease spread; incorporating household structure into ABMs enables in-depth study of within-household transmission dynamics and its overall impact at a population level, encompassing interactions like shared living spaces and close contacts that strongly shape disease transmission patterns [44].

Modelling TB in low and high-burden settings presents distinct challenges. Low-burden settings have lower TB incidence but better access to comprehensive data, potentially enabling greater precision in parameter estimation. However, TB transmission in these settings may be concentrated within high-risk groups, or, in certain cases, it’s so infrequent that it can be excluded altogether. In contrast, high-burden settings experience widespread transmission throughout the general population, often in crowded and high-risk environments, which makes it possible to identify broader epidemiological trends, but impossible to trace all transmission trees. In addition, the local healthcare infrastructures and socioeconomic elements vary, influencing the suitability and feasibility of intervention strategies. Low-burden contexts might concentrate on high-risk groups or specific regions, whereas high-burden contexts necessitate interventions encompassing the entire population. This divergence implies that parameter estimation from low-burden situations cannot be directly extrapolated to high-burden scenarios, although this may vary depending on the nature of the specific parameters (e.g. biological, social, programmatic).

The appropriateness of integrating economic evaluation within ABMs presents challenges due to their inherent complexity. The choice between ABMs and other modelling methods, such as system dynamics, semi-mechanistic techniques, and compartmental models, should be based on factors like the model’s scope and purpose, and the modeller’s expertise. Understanding the potential and limitations of ABMs in economic evaluation is essential for informed decision-making in disease modelling and cost-effectiveness analysis [45].

Calibration involves tuning parameters and rules to ensure the implemented model can align with real-world data, while also quantifying uncertainties in model parameter values. In the field of ABMs, achieving a deterministic equilibrium is precluded by their inherent stochastic nature [46]. Instead, ABMs find relative stability as agents interact over time, although even convergence to a pseudo-equilibrium may remain elusive, or not be representative of the historical dynamics of TB epidemiology. Hence, the burn-in period becomes essential for allowing emergent patterns to settle and stabilise within the system. The extended temporal scope demanded by the prolonged natural progression of TB introduces uncertainties in both modelling transmission dynamics and calibration. ABMs, therefore, need to capture historical dynamics spanning an extensive timeframe, encompassing diverse stages of TB infection, individual-level variations, and treatment outcomes. In this phase, the model’s trajectory is significantly influenced by randomness and variations in agent behaviour. The duration of the burn-in period is influenced by the complexity and stochastic nature of the model, established through sensitivity analyses to ensure results that are dependable and significant. In scenarios where the model aims to encapsulate the continual evolution of historical dynamics in an ever-shifting epidemiological backdrop, conventional burn-in periods might not apply. Instead, the focus could be on achieving a relatively stable “pseudo-equilibrium” offering a captivating insight highlighted in the review. Strategies like parallel computing are pivotal in managing this protracted timeframe, harmonising model complexity with clarity for practicality and effective communication of insights.

ABMs offer flexibility with diverse implementation tools, as highlighted in this review we observed the use of a diverse range of software tools. Simulation software, such as NetLogo or AnyLogic, offers user-friendly model construction, pre-built features, and visualisation convenience. However, they may have limitations in handling intricate analyses and customization. For intricate or specialised analyses, employing general programming languages like C++, Python, or Java can offer greater adaptability and integration with other commonly used libraries of these languages. Nonetheless, these languages demand higher upfront effort and programming expertise. The tool choice hinges on factors including problem complexity, modeller’s training, and project timeline, ensuring alignment with objectives and resources.

The efficiency of ABMs’ computational performance hinges on variables like agent quantity, interaction rule complexity, and hardware-software configurations. This diverges from deterministic models, where runtime is unaffected by population size. This accounts for individual-level variations in disease progression, improving accuracy in representing the natural history of TB. By directly modelling the distribution of sojourn times, ABMs can capture the variability and uncertainty in features such as the time it takes for latent TB infection to progress to active disease, incorporating factors like the host’s immune response and exposure levels. This adds complexity and realism to the model, making it a valuable alternative to the SEIR framework for TB research. In adapting compartmental models with stochastic elements, accurate replication of inherent variability is achieved, whether through stochastic differential equations or agent-based modelling. These simulations mirror empirical patterns and yield probability distributions for comparison, requiring careful attention to model assumptions, parameterization, and validation for accurate insights.

Defining a scale factor is a feasible and commonly used solution for managing computational expenses in ABMs. By judiciously adjusting this factor, specific simulation aspects can be simplified while preserving core dynamics. This balances computational efficiency and model accuracy, enabling the handling of extensive simulations or extended time frames while capturing essential emergent behaviours. In ABMs, establishing reasonable population scale factors requires a careful equilibrium between model precision and computational viability. The appropriateness of such a factor relies on considerations like data availability, research objectives, and detail level. While no universal rule exists, scaling factors that excessively distort the model’s scale beyond practical interactions should be avoided. Unrealistic scaling can misrepresent real-world dynamics, compromise efficiency, and skew simulation results.

Code reproducibility in ABM studies is crucial for scientific integrity. Among the 26 studies examined, only 8 provided publicly available code. Comprehensive model documentation, like the ODD protocol, was adhered to by only one study. Code reproducibility and transparency are crucial in ABM for to enable result validation, knowledge building, and collaboration. Sharing accessible code and comprehensive model documentation is imperative for scientific rigor and robustness, as it ensures replicability and verification, ultimately enhancing research credibility. Moreover, as tuberculosis models often inform public health policy, this practice is crucial for translating research into actionable policy decisions. Additionally, by sharing code and adhering to established documentation standards, researchers not only benefit their own work but also contribute to the broader development and improvement of modelling methodologies. This collaborative approach fosters innovation and the evolution of modelling techniques and practices.

Moving forward, there are several key areas for improvement in using ABMs for studying TB transmission. These include enhancing data availability and validation, integrating spatial and social heterogeneities, considering economic evaluation, refining calibration techniques and uncertainty analysis, improving computational efficiency, emphasising documentation and transparency of ABMs.

## Conclusions

In summary, ABMs offer a versatile and promising approach to understanding complex disease transmission, particularly relevant in cases like TB. Their capacity to capture heterogeneous mixing, often overlooked in traditional models, allows a more accurate representation of real-world dynamics. In the context of TB, this becomes particularly significant, as ABMs offer a unique opportunity to model and understand the importance of household transmission, which plays a pivotal role in the spread of the disease. However, advanced capabilities in agent-based models (ABMs) come with complexity, due to unobserved local interactions, parameter tuning, and high computational costs for simulating large populations. Improving computational efficiency is promising, but calibrating these models remains a significant challenge, primarily due to their inherent stochasticity. To enhance transparency and reproducibility in ABM, we actively endorse open-source code sharing and the adoption of standardised reporting. This proactive approach should strengthen ABMs’ reliability and impact in understanding complex disease dynamics like TB.

## Supplementary Information

Supplementary Material 1.

Supplementary Material 2.

Supplementary Material 3.

Supplementary Material 4.

## Data Availability

Data is provided within the manuscript or supplementary information files.

## Abbreviations

ABM: Agent-based modelling
HIV: Human immunodeficiency virus
ICERs: Incremental cost-effectiveness ratios
IBM: Individual-based Model
LTBI: Latent TB infection
*M.tb*: *Mycobacterium tuberculosis*
ODD: Overview, Design Concepts, and Details
PRISMA: Preferred Reporting Items for Systematic Reviews and Meta-Analyses
SEIR: Susceptible - Exposed - Infected – Recovered
TB: Tuberculosis
YLS: Year of life saved
