# Correction: Agent-based modelling of *Mycobacterium tuberculosis* transmission: a systematic review

**DOI:** 10.1186/s12879-026-13026-x

**Published:** 2026-03-24

**Authors:** Viet Long Bui, Angus E. Hughes, Romain Ragonnet, Michael T. Meehan, Alec Henderson, Emma S. McBryde, James M. Trauer

**Affiliations:** 1 School of Public Health and Preventive Medicine, Monash University, Melbourne, VIC Australia; 2 Australian Institute of Tropical Health and Medicine, James Cook University, Townsville, QLD Australia

**Correction: BMC Infectious Diseases (2024) 24:1394**

10.1186/s12879-024-10245-y

In Table 2 of this article, entries were incorrect and should have been.

**Table 2:** Characteristics of the reviewed studies

Characteristics	*N*	%
**TB Burden** ^**1**^		
High	11	42%
Moderate	1	4
Low	12	46%
Unclear	2	8%
**Heterogeneous mixing**		
Yes	15	58%
No	11	42%
**Age explicitly modelled**		
Yes	12	46%
No	14	54%
**Household structure represented**		
Yes	8	31%
No	18	69%
**Latent TB represented**		
Yes	23	88%
No	3	12%
**Spatial structure**		
Yes	5	18%
No	21	82%
**Economic evaluation**		
Yes	2	8%
No	24	92%
**Software**		
Simulation software^2^	12	50%
Self-developed^3^	9	32%
Not Declared	5	18%
**Code publicly accessible**		
Yes	8	29%
No	18	71%
**Total**	26	100%

^1^ As reported by authors

^2^ AnyLogic, NetLogo, EMOD, DTK)

^3^ MATLAB®, C/C++, Python, Go, Julia, etc...

The original article has been corrected.

