# Supplementary material for: Agent-based modelling of Mycobacterium tuberculosis transmission: a systematic review
Source: BMC Infect Dis. 2024 Dec 6;24:1394. doi: 10.1186/s12879-024-10245-y (PMC11622501; doi:10.1186/s12879-024-10245-y)
Supplement: Supplementary file 3 — Supplementary Material 3. [file 12879_2024_10245_MOESM3_ESM.pdf]

APPENDIX 3: SEARCH STRATEGIES

Database searches of OVID Medline, OVID Embase, Ovid Global Health and Scopus will be performed.

The search strategy aimed to return publications referring to each of the following three concepts in their subject heading, keywords list, title or abstract:

1. Terms relating to tuberculosis

2. Terms relating to epidemiology, demography, infectious disease outbreaks or transmission,

epidemics.

3.Terms relating to agent-based, individual-based or microsimulation models.

EMBASE:

1. exp tuberculosis/

2. (tuberculos* or tb).mp.

3. 1 or 2

4. exp epidemiological data/

5. exp epidemiological monitoring/

6. exp demography/

7. exp epidemic/

8. endemic disease/

9. pandemic/

10. epidemiolo*.mp.

11. disease outbreak*.mp.

12. disease transmis*.mp.

13. endemic*.mp.

14. epidemic*.mp.

15. pandemic*.mp.

16. spread*.mp.

17. 4 or 5 or 6 or 7 or 8 or 9 or 10 or 11 or 12 or 13 or 14 or 15 or 16

18. ((agent based or individual based or individual level or multi-agent or actor-based or network or stochastic or simulation* or microsimulation* or micro-simulation* or mechanistic or comput*) adj2 model*).mp.

19. 3 and 17 and 18

MEDLINE:

1. exp tuberculosis/ or mycobaterium tuberculosis/

2. (tuberculos* or tb).mp.

3. 1 or 2

4. exp epidemiology/

5. demography/

6. exp disease outbreaks/

7. exp disease transmission, infectious/

8. exp epidemiologic measurements/

9. exp epidemiologic methods/

10. epidemiolo*.mp.

11. disease outbreak*.mp.

12. disease transmis*.mp.

13. endemic*.mp.

14. epidemic*.mp.

15. pandemic*.mp.

16. spread*.mp.

17. 4 or 5 or 6 or 7 or 8 or 9 or 10 or 11 or 12 or 13 or 14 or 15 or 16

18. ((agent based or individual based or individual level or multi-agent or actor-based or network or stochastic or simulation* or microsimulation* or micro-simulation* or mechanistic or comput*) adj2 model*).mp.

19. 3 and 17 and 18

GLOBAL HEALTH:

1. exp tuberculosis/ or exp Mycobacterium tuberculosis/

2. (tuberculos* or tb).mp.

3. 1 or 2

4. exp epidemiology/

5. demography/

6. exp epidemics/

7. disease control/

8. disease transmission.mp. or transmission/ [mp=abstract, title, original title, heading words, cabicodes words]
9. epidemiolo*.mp.
10. disease outbreak*.mp.
11. disease transmis*.mp.
12. endemic*.mp.
13. epidemic*.mp.
14. pandemic*.mp.
15. spread*.mp.
16. 4 or 5 or 6 or 7 or 8 or 9 or 10 or 11 or 12 or 13 or 14 or 15
17. ((agent based or individual based or individual level or multi-agent or actor-based or network or stochastic or simulation* or microsimulation* or micro-simulation* or mechanistic or comput*) adj2 model*).mp.
18. 3 and 16 and 17

SCOPUS:

TITLE-ABS-KEY (tuberculosis OR tb) AND TITLE-ABS-KEY(epidemiolo* OR "disease outbreak*" OR "disease transmi*" OR dynamic* or spread*) AND TITLE-ABS-KEY(("agent based" OR "individual based" OR "individual level" OR "multi agent" OR "actor based" OR network OR stochastic OR simulation* OR microsimulation* OR micro-simulation* OR comput* OR mechanistic) W/1 model*)
